# Interlocking endoscopic sleeve gastroplasty with next-generation suturing device

**DOI:** 10.1016/j.vgie.2024.10.007

**Published:** 2024-10-11

**Authors:** Barham K. Abu Dayyeh

**Affiliations:** Division of Gastroenterology and Hepatology, Mayo Clinic, Rochester, Minnesota, USA

## Background

**Figure 1:**
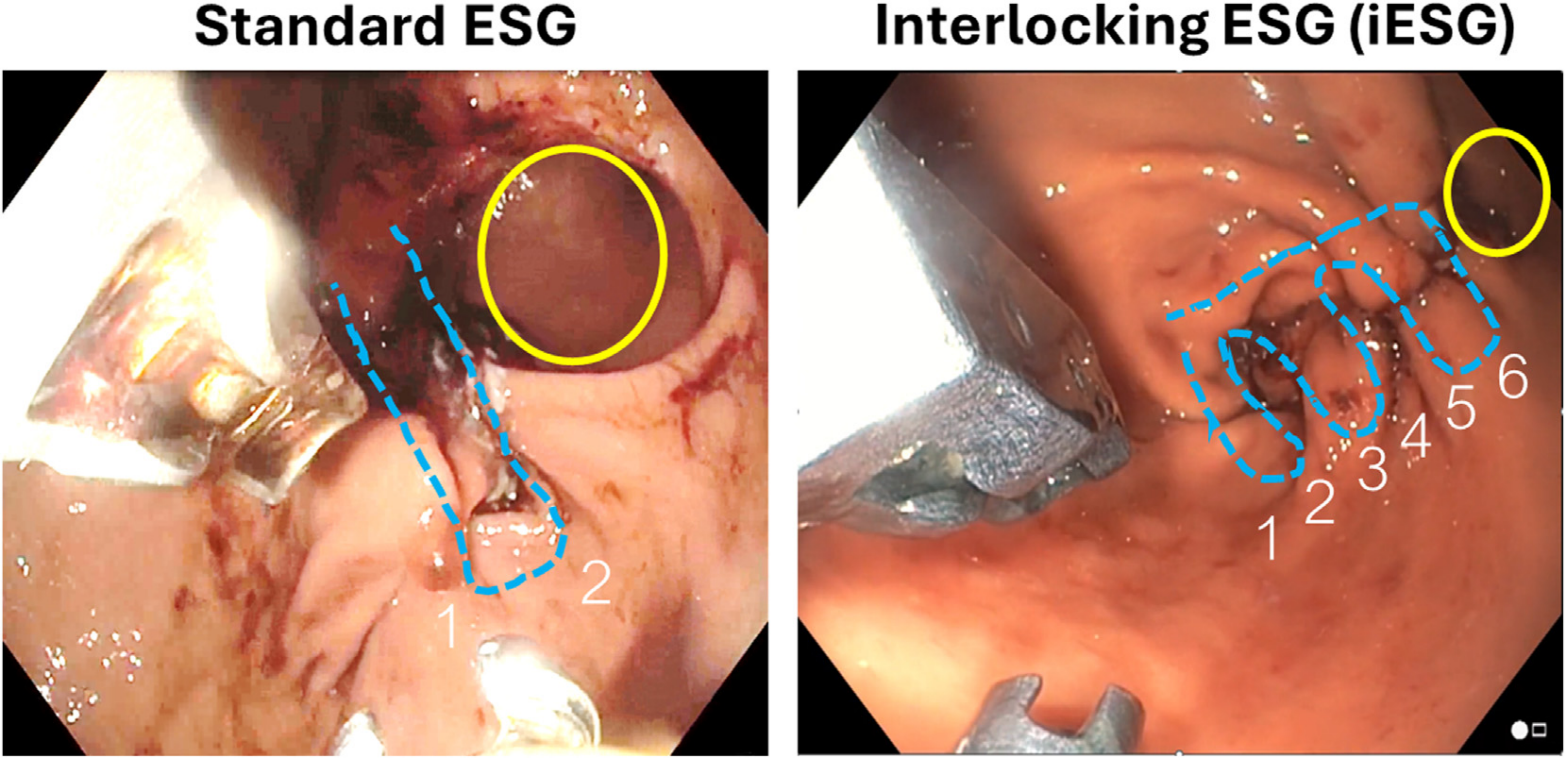
Comparison of the plication constructs in standard ESG versus iESG, highlighting the interlocking pattern achieved with 6 layers of staggered sutures in iESG, compared with the traditional 2-layered U-shaped sequence. Note the narrower endoscopic sleeve lumen in the iESG construct, as indicated by the *yellow circle*. *ESG*, Endoscopic sleeve gastroplasty; *iESG*, interlocking endoscopic sleeve gastroplasty.

Endoscopic sleeve gastroplasty (ESG) is a minimally invasive, organ-preserving, and cost-effective weight loss procedure designed for patients who are either ineligible for surgery or prefer a less invasive alternative.^1^, ^2^, ^3^, ^4^ The traditional ESG technique involves the use of a suturing device mounted on a double-channel gastroscope to place U-shaped sutures at each plication sequence. Although effective, this approach presents certain limitations: the U-shaped sutures approximate the plicated surfaces without maximizing surface-area contact, and the looping of the suture line during placement hinders the formation of a multilayered gastroplasty sequence (Fig. 1). In this video, we introduce an interlocking suturing pattern for ESG using a next-generation suturing device. This novel technique enhances both the efficiency and durability of the procedure while reducing the total number of sutures required compared with the traditional method.

## Method

In this demonstration (Video 1, available online at www.videogie.org), we used the next-generation OverStitch NXT Endoscopic Suturing System (Boston Scientific, Marlborough, Mass, USA), which is compatible with a single-channel gastroscope and offers several key technical advantages for performing the interlocking endoscopic sleeve gastroplasty (iESG) procedure. This system is equipped with a physician-controlled helical retractor that aligns with the suturing arm, enabling larger and more consistent full-thickness plications. The suturing arm's improved bending angle and rotational capabilities allow for precise articulation, even in challenging anatomical positions. Additionally, the system includes an auxiliary channel that provides superior suction and irrigation, ensuring optimal visualization and a clear operative field throughout the procedure.

The iESG sequence begins with the application of 2 layers of staggered sutures, creating interlocking peaks and valleys that maximize surface contact between the approximated gastric walls. This initial layer is followed by the continuous application of 4 additional staggered layers of 2-0 Prolene sutures (Boston Scientific, Marlborough, Mass, USA), which further interlock the plication sequence, tubularize the stomach, and reduce its longitudinal length at each sequence. In the case presented, involving a patient with class II obesity, the iESG procedure was successfully completed with only 5 sutures and cinches.

**Figure 2:**
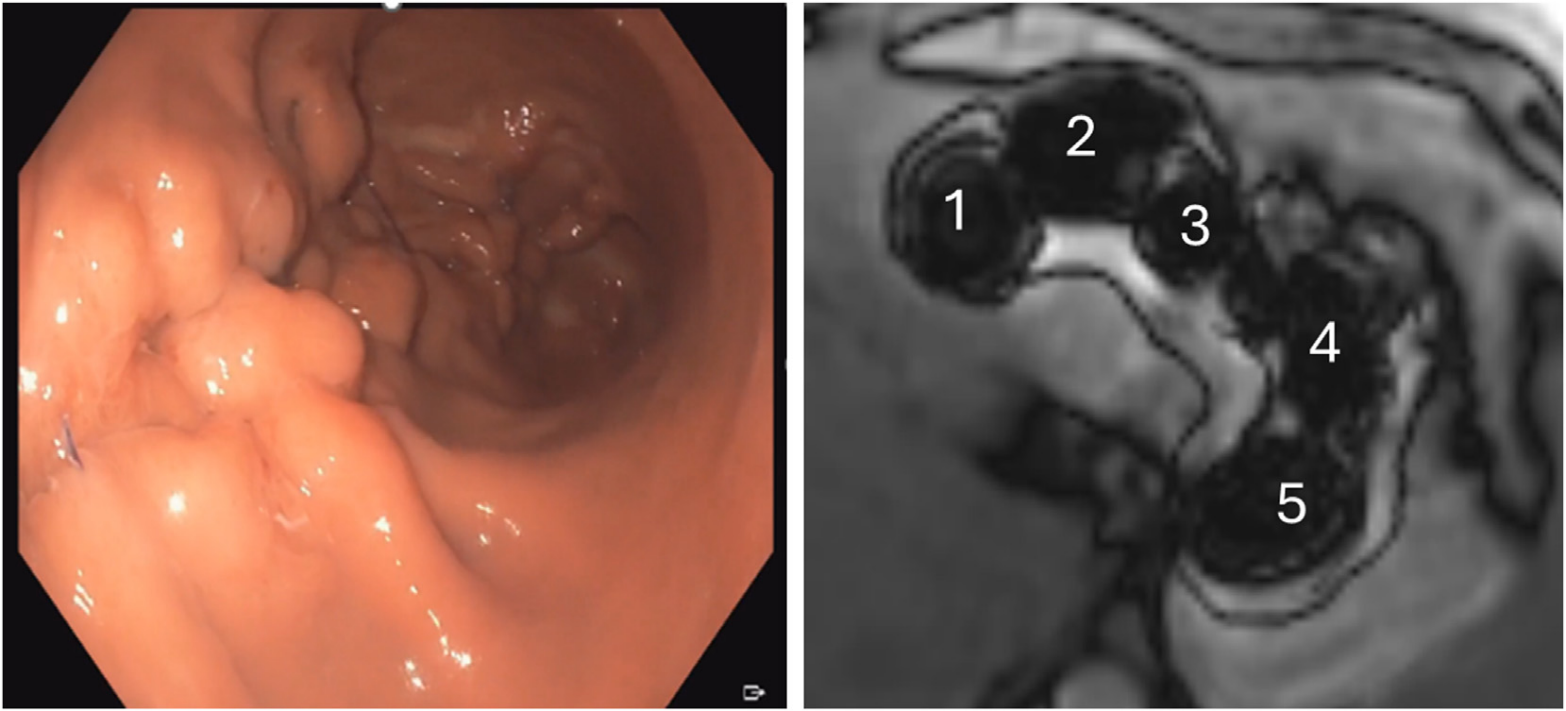
Endoscopic and magnetic resonance imaging evaluation of the iESG at 6 months demonstrating full retention of all sutures, robust full-thickness plications, and maintenance of the endoscopic sleeve effect. *iESG*, Interlocking endoscopic sleeve gastroplasty.

The durability of the iESG was assessed at 6 months postprocedure using endoscopy and oral contrast-enhanced magnetic resonance imaging (Fig. 2), which confirmed the full retention of all sutures, robust plications, and the maintenance of the endoscopic sleeve effect. The patient achieved a 23% total body weight loss at 6 months, with no reported adverse events. A larger clinical trial comparing the outcomes of iESG versus the standard ESG approach is currently underway.

## Conclusion

Interlocking ESG (iESG) using a next-generation endoscopic suturing device represents a significant advancement in ESG, offering improvements in both procedural efficiency and durability. This technique has the potential to enhance clinical outcomes and expand the applicability of ESG in the management of obesity.

## Disclosures

Dr Abu Dayyeh is a consultant for Boston Scientific, Medtronic, Apollo Endosurgery, and Olympus; receives research support from Boston Scientific, Medtronic, Apollo Endosurgery, and USGI Medical; and is a coinventor of the Endogenex technology licensed by Mayo Clinic, with institutional equity and royalty through Mayo Clinic's invention policy.

## Supplementary data

Video 1 Demonstration of the interlocking endoscopic sleeve gastroplasty (iESG) procedure using a next-generation endoscopic suturing device.
